# Stopping eyes and hands: evidence for non-independence of stop and go processes and for a separation of central and peripheral inhibition

**DOI:** 10.3389/fnhum.2014.00061

**Published:** 2014-02-18

**Authors:** Alessandro Gulberti, Petra A. Arndt, Hans Colonius

**Affiliations:** ^1^Department of Neurophysiology and Pathophysiology, University Medical Center Hamburg-EppendorfHamburg, Germany; ^2^Transferzentrum für Neurowissenschaften und LernenUlm, Germany; ^3^Department of Psychology and Cluster of Excellence Hearing4all, University of OldenburgOldenburg, Germany

**Keywords:** stop signal, race model, inhibitory control, eye-hand coordination, hand movements, saccadic reaction time

## Abstract

In the stop-signal paradigm, participants perform a primary reaction task, for example a visual or auditory discrimination task, and have to react to a go stimulus as quickly as possible with a specified motor response. In a certain percentage of trials, after presentation of the stimulus (go signal), another stimulus (stop signal) is presented with a variable stop-signal delay. Whenever a stop signal occurs, the participant is asked to inhibit the execution of the response. Here, an extended test of the popular horse race model for this task (Logan and Cowan, 1984) is presented. Responses for eye and hand movements in both single-task and dual-task conditions were collected. Saccadic reaction times revealed some significant violations of the model's basic assumption of independent go and inhibition processes for all six participants. Saccades that escaped an early stop signal were systematically slower and had smaller amplitudes compared to saccades without a stop signal. Moreover, the analysis of concomitant electromyographic responses recorded from the upper arm suggests the existence of two separate inhibitory mechanisms: a slow, selective, central inhibitory mechanism and a faster, highly efficient, peripheral one, which is probably ineffective for saccades.

## Introduction

Stopping the execution of a planned movement, or suddenly interrupting an ongoing action to perform an alternative one are the key functions of inhibition. In order to explain inhibition processes, the existence of an overarching system that controls and coordinates different cognitive mechanisms has been hypothesized (Baddeley, 1986; De Jong et al., 1990; Burgess, 1997). More specifically, two separate inhibition mechanisms, a slow and selective central mechanism, and a quick and highly efficient peripheral mechanism, have been suggested (Bullock and Grossberg, 1988; De Jong et al., 1990). Here, these mechanisms are further explored by examining the inhibition of saccadic, manual, and muscle responses in a well-trained task.

Eye movements offer considerable potential for exploring movement inhibition, thanks to an extensive knowledge of the neuronal pathways that trigger and guide these movements (Schall, 1995; Findlay and Walker, 1999; Schall and Godlove, 2012). The neural control of saccades allows a limited number of movement types determined by three complementary muscle pairs (Goldberg et al., 1991). The fact that saccades are ballistic, that is, an activation command to the eye muscles cannot be stopped by a sudden activation of the antagonist muscles, offers a unique possibility to study performance of the slow and selective central inhibition mechanism since they cannot be stopped by a putative quick peripheral inhibition mechanism (Goldberg et al., 1991; Hanes and Schall, 1995; Cabel et al., 2000). In contrast, manual pointing movements (executed by raising the forearms) can be stopped by the activation of antagonist muscles. Thus, if the execution of a planned pointing movement has been inhibited, recording of the *biceps brachii* reveals muscular activation even when the corresponding index finger has not been removed from the sensor used for the detection of pointing movements. This combination gives a unique opportunity to discriminate the two presumably separate inhibition mechanisms and to disentangle their impact on the performance of different effectors.

The stop-signal paradigm is arguably the most common behavioral method to explore inhibition (Verbruggen and Logan, 2008). In this paradigm, participants perform a primary reaction task, for example a visual or auditory discrimination task, and they have to react to a given stimulus as quickly as possible with a given motor response (Ollman, 1973). In a certain percentage of trials, after presentation of the stimulus (go signal) a stop signal will be presented after a variable stop-signal delay (SSD). Whenever a stop signal occurs, the participant is asked to try to inhibit the execution of the response action already being planned.

The processes resulting from an inhibition mechanism are only partially quantifiable (Colonius, 1990). In particular, if an action is successfully inhibited, no observable response can be recorded except for the frequency of this event happening. Notably, the stop-signal paradigm involves a set of dependent variables that can be measured, i.e., whether the participant responds to the primary task stimulus (go signal), what time took the response after onset of the go signal, and how often the participant was able to inhibit the primary reaction.

**Figure 1:**
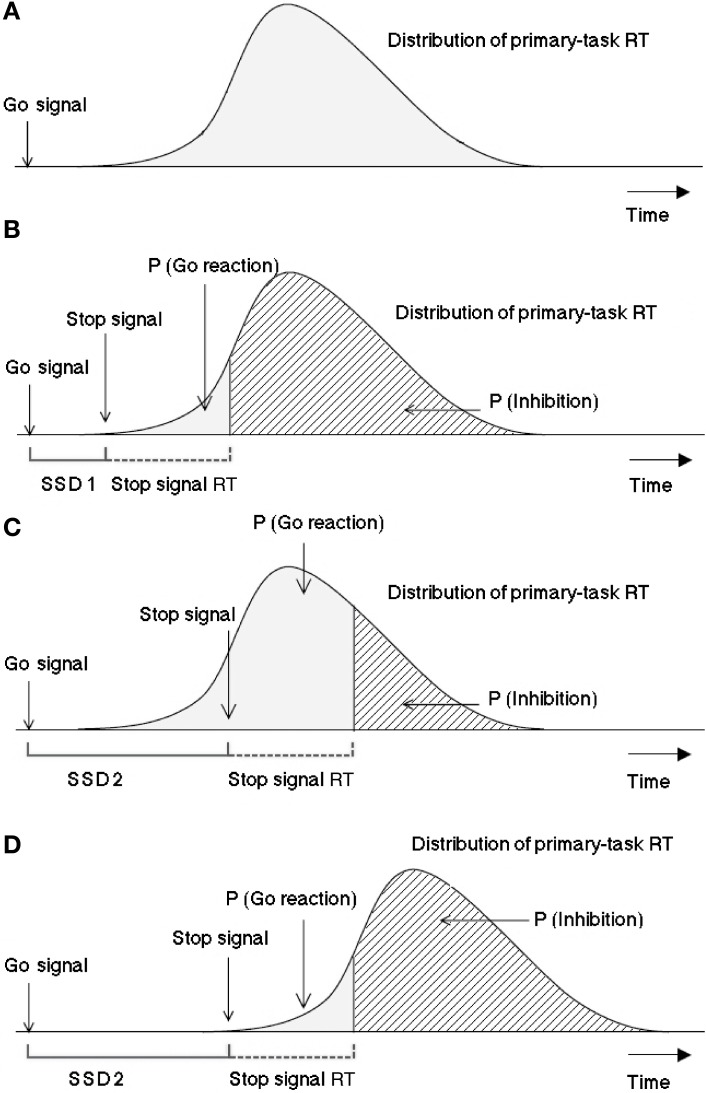
**Graphic representation of the assumptions of the Logan-Cowan horse race model indicating the probability of responding given a stop signal and the probability of inhibition depending on SSDs**. The first panel **(A)** is a histogram of response times (RT) distribution for the primary task. In 25% of the cases after a varying time interval (SSD 1) a stop signal is presented, as depicted in panel **(B)**. The processing of the stop signal needs a certain amount of time, which is considered by the model to be constant. The third panel **(C)** visualizes the case in which the stop signal is presented later after the go signal (SSD 2) in respect to SSD 1. In this case, assumed that the stop-signal reaction time (SSRT) and the distribution of the reaction times to the primary task remain the same, the probability for the go reaction to win the race would be higher and that to stop lower. The last panel **(D)** shows the effect of a later presentation of the stop signal with respect to a distribution which has been shifted to the right (i.e., in the case of a task requiring longer processing or execution, like hand responses in comparison to saccadic reactions). This case exhibits the same probability for the go signals to win the race as in the panel **(B)** within the horse race model not only the latencies between go and stop signals determine the inhibition probability, but also the time needed for the go and stop signals to be processed should be considered. Adapted from Logan and Cowan (1984).

Logan and Cowan (1984) suggested the so-called “horse race model” to explore inhibition mechanisms: Processes triggered by the go signal and by the stop signal are independent and compete against each other over time. The process with the fastest termination time wins the race and blocks the effect of the second process completely. Figure 1 depicts a graphic representation of the model.

The earlier a stop signal is presented, the more likely it is the go-signal reaction will be canceled. The later a stop signal occurs, the more likely it is for the go process to win the race against the inhibition process and, thus, for a response to be executed. For every given SSD, the response probability can be estimated from the observed relative frequency of responses. This response frequency corresponds to the proportion of go processes ending before the inhibition process and can be observed directly. Stop-signal reaction time^1^ (SSRT), that is, the time required to cancel the response being programmed, can be estimated as follows. Assuming SSRT to be constant, Logan and Cowan (1984) showed that the mean SSRT is equal to the difference between the point at which the stop signal was presented and the point at which the stopping process finished. The latter point can be estimated from the observed distribution function of responses when no stop signal was presented, by integrating it until the area under the integral equals the probability of responding (integration method). Although the assumption of constant SSRT is unlikely to hold strictly, this procedure has been shown to yield stable estimates for SSRT under most circumstances (De Jong et al., 1990; but see Verbruggen et al., 2013), and SSRT has become a prime measure of cognitive control. Three empirically testable predictions follow from the horse race model:

First Prediction: Reaction times of stop failures, that is, responses that escape inhibition (stop-failure RTs, for short), should be faster than go-signal reaction times, that is, responses to the go signal when no stop signal is present (go RTs for short).
This follows from stop-failure RTs coming from the same distribution as go RTs except that the stopping process cuts off the upper tail (cf. Figure 1).
Second Prediction: Mean stop-failure RT should increase as SSD increases, approximating the mean go RT.
This follows because slower responses win the race when the stop signal is delayed further.
Third Prediction: For any given SSD, mean stop-failure RT should equal the mean of those go RTs that are faster than the sum of the estimated SSRT and the SSD.

Accordingly, the three last panels of Figure 1 suggest a method to predict mean stop-failure RT by averaging over response times occurring to the left of the finishing time of the SSRT process plus SSD. Typically, satisfactory predictions can only be obtained for longer SSDs where stop-failure responses are more numerous than for shorter SSDs.

All three predictions have been confirmed many times both for manual and saccadic responses, therewith lending considerable support to the horse race model (Logan, 1981; Logan and Cowan, 1984; Osman et al., 1986; De Jong et al., 1990; Boucher et al., 2007b). Furthermore, strong support for the validity of the race model was provided by neurophysiological studies, demonstrating that the estimated SSRT based on the race model matches the timing of rising or dropping neuronal activity in a saccadic countermanding task (Hanes et al., 1998; Schall and Boucher, 2007). More recently, however, a number of investigations have challenged some of the underlying assumptions of the horse race model, in particular (i) the assumption of independence of the go and the stop processes (e.g., Oezyurt et al., 2003; Nelson et al., 2010) and (ii) the assumption of independence between trials, that is, stationarity of the responses across trials (Emeric et al., 2007; Bissett and Logan, 2012a,b). Only the first issue is of concern in this article. These recent reports of violations of the model premises, in particular for eyes-stop performances (Colonius et al., 2001; Oezyurt et al., 2003; Akerfelt et al., 2006), encouraged the further exploration of these phenomena in the present experiment. For these reasons, we made the conscious choice for an intensive long time experiment with six participants, where a long training session and consequent feedback played an important role. The aim was to collect enough data to reach statistical significance also at the shortest SSDs, where the violations have usually been observed.

Neurophysiological studies with saccadic eye movements have overwhelmingly demonstrated that saccades are produced by a network of mutually inhibitory gaze-shifting and gaze-holding neurons (Hanes et al., 1998; Paré and Hanes, 2003; see Boucher et al., 2007a for an overview). The question is how such interacting neural units can produce behavior that, according to the Logan-Cowan horse race model, should be the outcome of a race between processes with independent finishing times? The issue is an important one, since the validity of SSRT as a ubiquitous measure of cognitive control derives entirely from the validity of the independent race model (Boucher et al., 2007b). In this vein, Boucher et al. (2007a) proposed an interactive race model, assuming that the stop and go processes are independent for most of their duration. In a second stage, then, the stop process interacts strongly and briefly to interrupt the go process.

Here we re-tested the original model's basic assumption of independent go and inhibition processes, paying particular attention to fast reactions escaping early SSDs. Saccadic, manual, and biceps responses can all be subjected to the race model predictions listed above. Comparing inhibition probabilities and SSRTs across these different response modes should also be informative with respect to the issue of a central vs. a peripheral inhibition process.

## Methods

### Participants

Six student volunteers (5 female, mean age = 27 years, *SD* = 10 years) participated in the experiment after giving informed consent. All reported having normal hearing and normal or corrected-to-normal vision. Every participant was tested for eye and hand dominance. They were compensated partly with academic credits and partly with money paid after the last experimental session. The experiment was approved by the ethics committee of the University of Oldenburg, in accordance with the ethical standards of the 1964 Declaration of Helsinki.

### Apparatus and stimuli

Participants were seated in a darkened, sound-attenuated chamber (1.0 × 1.2 × 1.9 m). Their elbows and hands were positioned on a board in front of them and their head on a chin rest mounted on the board. Both index fingers were positioned on two photoelectric sensors (TCRT 100, Vishay Telefunken, operating wavelength 950 nm) mounted on the board in front of the chin rest. The distance between the sensors and the midline was 4.25 cm to the left and to the right. On a 37-inch monitor (XP37, NEC, image frequency 75 Hz), at a viewing distance of 57 cm, white dots (diameter 0.1°; luminance 19.8 cd/m^2^) were presented as visual stimuli against a dark background (intensity below 0.01 cd/m^2^). Their eccentricity to the right or the left of the central fixation point was 25°. As an auditory stimulus, a noise signal (bandwidth 500–14000 Hz, intensity 77 dB SPL) was presented for 500 ms via headphones (Sennheiser HD 580). The noise signal had been convolved with a head-related transfer function of a dummy head such that the signal appeared to be located straight in front of the participant. One PC controlled the presentation of both the visual and auditory stimuli. A second PC was used for data recording.

### Experimental paradigm

**Figure 2:**
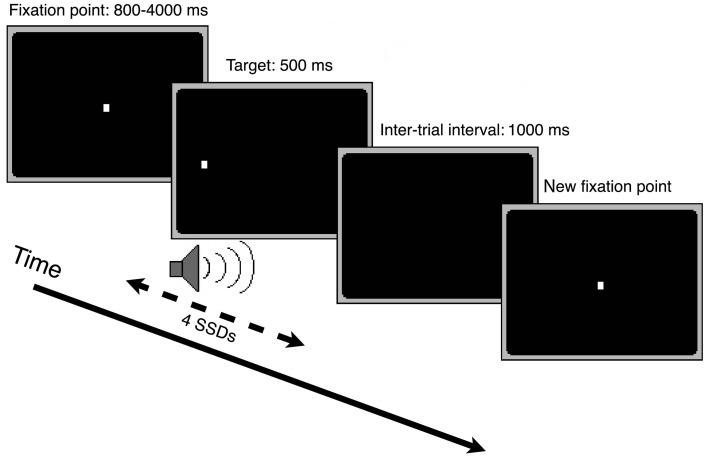
**Depiction of the course of one trial in the stop-signal paradigm**.

We examined the inhibition of saccadic, manual, and muscle responses in an intensively trained, multiple-session experiment. Participants came to the laboratory 1–2 times a week and performed only one session per day. Sessions consisted of three different condition blocks of 128 trials each (“oculomotor only,” “manual only,” and “both” condition block). Each trial started with the presentation of a central fixation point. After an interval varying randomly between 800 and 4000 ms, the fixation point disappeared and an identical target point appeared immediately after, evoking a phi phenomenon of the light dot. The participants had to perform a saccade, a manual pointing movement, or both in the direction of the target stimulus appearing randomly left or right. After 500 ms the target disappeared. After an interval of 1000 ms the next trial started (see Figure 2).

Pointing movements to targets in the left hemifield were made by raising the left forearm and index finger and those to targets in the right hemifield by raising the right forearm and index finger. In all condition blocks, the movement(s) had to be performed as quickly as possible after stimulus onset. In the “manual only” condition block, it was important that participants maintained fixation on the center of the screen when the fixation point disappeared. The order of the three condition blocks was counterbalanced between sessions.

Twenty-five percent of the 128 trials (32 trials per condition block) were stop trials, that is, in addition to the visual target, an auditory stop signal was presented for 500 ms. In this case, participants were asked to try to inhibit any response to the visual stimulus. The stop signals were given with four possible signal delays (SSDs) with respect to the target onset.

Participants were instructed that the most important task was to react to the visual targets as quickly as possible, however, without ignoring the auditory stop signals. Importantly, they were explicitly instructed not to delay their reactions to facilitate movement inhibition during a stop trial.

### Training and response-rate tracking procedure^2^

**Figure 3:**
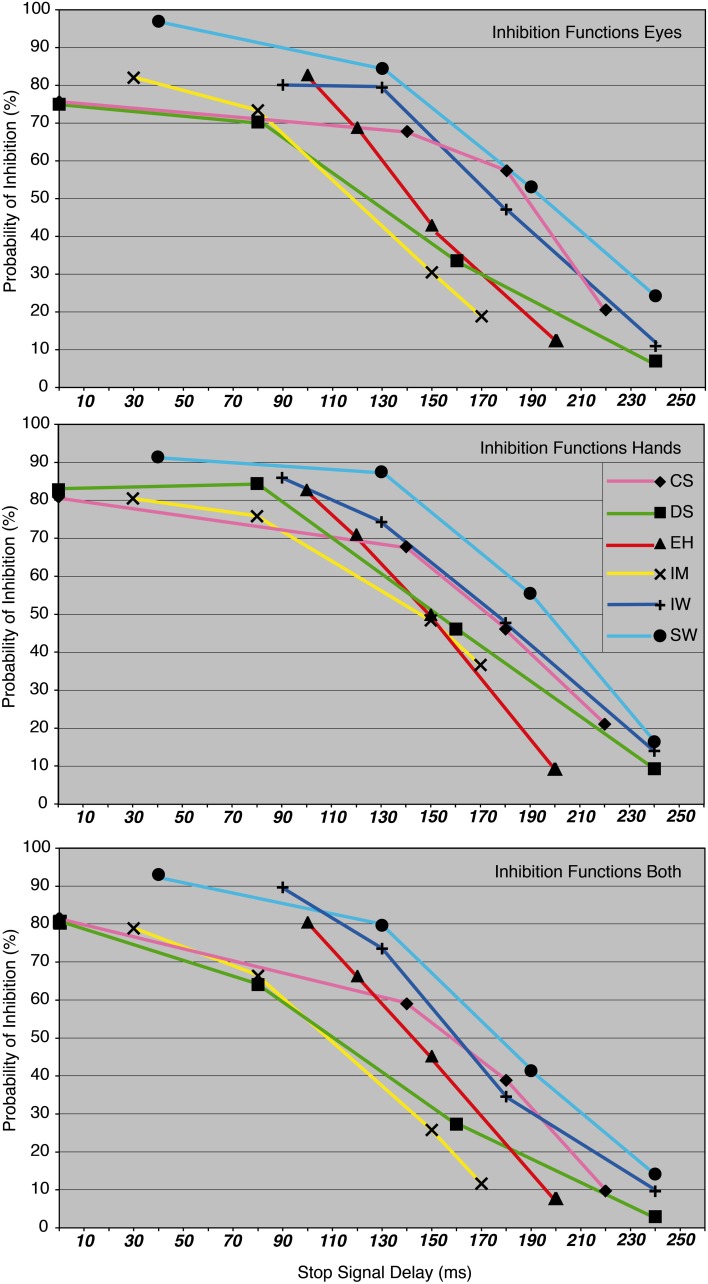
**The probability of inhibition in stop trials is plotted as a function of the stop-signal delay for eye, hand, and dual task, for each participant**.

Before beginning with the main experiment, each participant carried out an intensive training phase. The aim of the training and response-rate tracking phase was to determine individual SSDs for the three condition blocks. About 10–12 training sessions were required for each participant in order to stabilize their performance at approximately 50% of successfully inhibited responses. At the same time, four individual SSDs were determined for each of the participants in order to obtain inhibition probabilities of approximately 90, 70, 40, and 20% for the main experiment (see Figure 3 and Table 1).

**Table 1:** **The four SSDs (ms) were individually set to obtain inhibition probabilities of approximately 90, 70, 40, and 20% for each of the participants**.

	**SSD 1 inhib. prob. 90%**	**SSD 2 inhib. prob. 70%**	**SSD 3 inhib. prob. 40%**	**SSD 4 inhib. prob. 20%**
CS	0	140	180	220
DS	0	80	160	240
EH	100	120	150	200
IM	30	80	150	170
IW	90	130	180	240
SW	40	130	190	240

After the training phase, each participant performed at least 48 blocks over 16 experimental sessions. Table 2 shows the amount of data recorded for this experiment and also reports the number of trials collected per condition block. Each of the four SSDs was presented eight times in each condition block. In our experiment at least 16 valid condition blocks for each participant were evaluated, resulting in 128 stop trials per SSD. Horse race model simulations (Band et al., 2003) have shown that 40–70 trials per SSD, using the response-rate-tracking procedure, are required to attain a reliable estimate of inhibition (95% confidence interval) for all SSDs. Given this, a sufficient amount of data was collected for the estimation of SSRTs (Table 2). Nevertheless, it is important to mention that the sample size is quite different for stop-failure responses at the four SSDs. This fact may lead to a bias, when comparing stop-failure RT distributions at different SSDs.

**Table 2:** **Number of experimental sessions performed and total of single trials recorded for each of the six participants (training phase sessions are excluded from this count)**.

	**CS**	**DS**	**EH**	**IM**	**IW**	**SW**
Performed sessions	19	16	16	16	17	16
Total trials	7168	6144	6144	6144	6528	6144
Stop trials (25% of total trials)	1792	1536	1536	1536	1632	1536
Stop trials per condition block	608	512	512	512	544	512

Twenty-five percent of the trials were stop trials subdivided into the three condition blocks: “oculomotor only,” “manual only,” and “both”.

### Response recording and detection

Eye movements were recorded with an infrared light reflecting system (IRIS, Skalar Medicals). This system provided an analog signal of the eye position with a maximal spatial resolution of 0.03°. The eye position signal was digitized at a rate of 1 kHz and stored for offline analysis. Saccade onsets and offsets were detected automatically using the criteria: velocity of (a) more than 60° per second for onset, and (b) 15° per second for offset. The accuracy of the automatic detection was verified by visual inspection of the data. Saccades in the target direction with amplitude of more than 3° and latency between 80 and 600 ms were considered a valid response. Trials containing blinks, micro-saccades or drifts larger than 3° during fixation were excluded from further analysis. For each participant, only the dominant eye was analyzed (Becker, 1991; Oezyurt et al., 2003).

Finger movement was detected using photoelectric sensors giving a zero or one signal.

**Figure 4:**
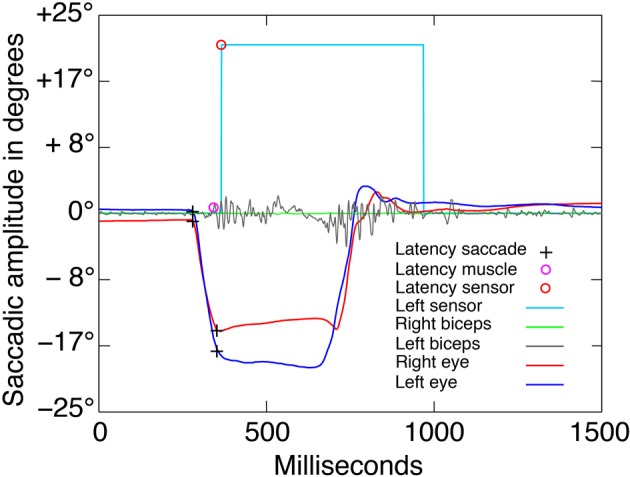
**Saccadic and EMG raw data**. Example of one go trial of the “both” condition block. The visual go signal was presented at latency 0 with an eccentricity of −25° to left with respect to the central fixation point. The black crosses indicate the saccade onsets and offsets, which were detected automatically using the criteria described in the methods. Only the dominant eye was analyzed. The violet and orange circles indicate the automatic detection of latencies from the left biceps EMG activity and the left finger photoelectric sensor, respectively. The accuracy of the automatic detection was verified by trial-by-trial visual inspection.

Arm muscle activity was measured with electromyographic (EMG) electrodes attached bilaterally to the *biceps brachii*. These signals were used to detect muscle activity indicating arm movement initiation even if the fingers were not removed from the sensors. It was therefore possible to detect motor commands that reached the musculature. A biceps reaction was considered a response to the target stimulus if it had an amplitude of 30 mV and occurred between 100 and 800 ms after target presentation (see Figure 4).

## Results

Values reported here are always mean or grand mean ± standard error of the mean (s.e.m.). *P* values refer always to paired-sample *t*-test, two-tailed, if not noted otherwise.

### Inhibition probabilities

Averaged over condition blocks, participants' inhibition probabilities for eye and hand movements were in the same range (grand mean for eyes: 56.2 ± 2.6% vs. grand mean for hands: 58.1 ± 1.7%, *p* = 0.44), although latencies differed markedly (see below). Across the entire sample, the inhibition probability for biceps activation was always smaller than the probability for the onset of the corresponding pointing movement (grand mean for biceps: 51.2 ± 2.1% vs. grand mean for hands: 58.1 ± 1.7%, *p* < 0.001). Participants CS, IW, and SW showed higher inhibition probabilities for saccades compared to pointing movements (*CS* = 60.6 vs. 56%, *IW* = 57 vs. 55.1%, *SW* = 65.9 vs. 65.4%).

No significant differences were found between inhibition functions in the single-response vs. dual-response condition for eyes (single: 55.7 ± 2.5% vs. dual: 56.7 ± 2.8%, *p* = 0.47), hands (single: 58.4 ± 1.8% vs. dual: 57.9 ± 1.7%, *p* = 0.66) and biceps activation (single: 50.8 ± 2.4% vs. dual: 51.5 ± 2.0%, *p* = 0.59).

Inhibition probabilities for saccades and pointing movements in the dual-response condition varied across subjects. This was because in some stop trials only the eye or the hand movement was inhibited successfully. The stopping of eye movements while the hand movement was executed occurred in 3.6–15.3% (mean = 6.9%) of the stop trials. The hand movement was stopped alone on average in 7.5% of the stop trials (range: 1.7–13.4%).

### Saccadic amplitudes

All participants showed larger saccadic amplitudes in go trials than in stop trials (stop failures; Table 3). This effect occurred in single-response as well as in dual-response conditions. Amplitudes in go trials were significantly larger than in stop failures for all participants except IM in the single-response condition (ANOVA: CS *p* < 0.001, DS *p* < 0.001, EH *p* < 0.001, IW *p* = 0.026, SW *p* < 0.001) and for four of the six participants in the dual-response condition (ANOVA: CS *p* < 0.001, DS *p* < 0.001, EH *p* = 0.002, SW *p* < 0.001). In the group statistic for the single-response condition, grand mean amplitudes of go trials (19 ± 0.3°) were significantly larger than grand mean amplitudes of stop failures (17.7 ± 0.6°, *p* = 0.009, paired-sample *t*-test, one-tailed^3^). For the dual-response condition, grand mean amplitudes of go trials (18.7 ± 0.3°) were also significantly larger than stop failures (17.9 ± 0.5°, *p* = 0.037, paired-sample *t*-test, one-tailed). Considering the saccadic responses of single and dual conditions together, go trials' amplitudes (18.9 ± 0.2°) were significantly larger than stop-failures' amplitudes (17.8 ± 0.4°, *p* < 0.001, paired-sample *t*-test, one-tailed). No significant differences for saccadic amplitudes were found in the comparison between single-response vs. dual-response conditions.

**Table 3:** **Comparison of saccadic amplitudes in go trials and stop trials for single-response and dual-response conditions**.

	**Single response**			**Dual response**		
	**Amplitude:**		***U*-test**	**Amplitude:**		***U*-test**
	**Go**	**Stop**	***p*(2-tailed)**	**Go**	**Stop**	***p*(2-tailed)**
CS	17.9 > 15.2°		*p* < 0.001	18.0 > 15.6°		*p* < 0.001
DS	19.0 > 17.0°		*p* < 0.001	18.8 > 18.0°		*p* < 0.001
EH	19.6 > 18.1°		*p* < 0.001	18.5 > 18.1°		*p* = 0.002
IM	19.6 > 19.3°		n.s.	19.8 > 19.8°		n.s.
IW	19.3 > 19.0°		*p* = 0.026	18.3 > 18.0°		n.s.
SW	18.7 > 17.5°		*p* < 0.001	18.7 > 17.9°		*p* < 0.001
	**Amplitude:**		***T*-test**	**Amplitude:**		***T*-test**
	**Go**	**Stop**	***p*(1-tailed)**	**Go**	**Stop**	***p*(1-tailed)**
GM	19.0 > 17.7°		*p* = 0.009	18.7 > 17.9°		*p* = 0.037

GM, grand mean.

### Testing the race model

#### Testing the first prediction: reaction times in stop trials and go trials

Reaction times were obtained for responses in go trials (go RTs) and for responses in stop trials (stop-failure RTs). According to the model, stop-failure RTs should be shorter than go RTs. To test this prediction, average go and stop-failure RTs in the three condition blocks were compared for each participant (Table 4).

**Table 4:** **Comparison of mean latencies in go trials and stop trials for the three condition blocks**.

		**Oculomotor**			**Manual**				**Both**		
		**Mean RT (ms):**		***U*-test**	**Mean RT (ms):**			***U*-test**	**Mean RT (ms):**		***U*-test**
		**Go**	**Stop**	***p*(2-tailed)**	**Go**	**Stop**		***p*(2-tailed)**	**Go**	**Stop**	***p*(2-tailed)**
CS	Eyes	294.4 **<** 302.5		0.898					289.6 > 289.2		0.132
	Hands				344.5 > 340.9			0.002	345.9 > 343.1		0.045
	Biceps				306.3 > 299.6			<0.001	308.1 > 305.4		0.001
DS	Eyes	243.9 > 238.9		0.060					249.1 > 240.9		0.072
	Hands				320.3 > 301.9			<0.001	326.0 > 305.3		<0.001
	Biceps				265.7 > 248.8			<0.001	272.8 > 256.3		<0.001
EH	Eyes	**242.6 < 252.3**		0.001					257.2 **<** 266.9		0.011
	Hands				331.0 > 313.5			<0.001	331.4 > 315.5		<0.001
	Biceps				263.2 > 250.3			<0.001	264.2 > 250.9		<0.001
IM	Eyes	**238.1 < 255.9**		<0.001					**242.7 < 263.8**		<0.001
	Hands				358.9 > 354.0			0.062	357.8 > 357.1		0.526
	Biceps				298.1 > 288.3			0.001	293.8 > 290.6		0.087
IW	Eyes	**263.2 < 278.3**		<0.001					280.0 **<** 283.4		0.929
	Hands				315.5 > 293.3			<0.001	314.6 > 292.9		<0.001
	Biceps				259.3 > 249.4			<0.001	261.0 > 249.4		<0.001
SW	Eyes	293.7 > 279.1		0.002					289.5 > 277.9		0.001
	Hands				374.3 > 346.0			<0.001	371.7 > 353.3		<0.001
	Biceps				323.1 > 294.6			<0.001	319.7 > 298.5		<0.001
		**Mean RT (ms)**		***T*-test**	**Mean RT (ms)**			***T*-test**	**Mean RT (ms)**		***T*-test**
		**Go**	**Stop**	***p*(2-tailed)**	**Go**	**Stop**		***p*(2-tailed)**	**Go**	**Stop**	***p*(2-tailed)**
GM	Eyes	262.6 **<** 267.8		0.356					268.0 **<** 270.3		0.654
	Hands				340.7 > 324.9			0.010	341.2 > 327.9		0.020
	Biceps				285.9 > 271.8			0.007	286.6 > 275.2		0.010

Marked in bold are differences between go RTs and stop-failure RTs, which showed a trend opposite to the predictions. GM, grand mean.

In the condition block “manual only,” the hand and biceps reactions generally matched the model predictions. Only the hand reactions of participant IM did not reach significant values.

Numerous discrepancies with the model predictions were found for saccadic eye movements in the condition block “oculomotor only,” however. For participants EH, IM, and IW saccadic RTs in stop trials were significantly longer than in trials without stop signal. This indicates a significant lengthening of oculomotor processing time in trials in which a stop signal is presented. Only participant SW exhibited significantly shorter stop-failure RTs than go RTs. Saccadic RTs in block “both” were generally longer compared to those in block “oculomotor only,” but only participant IM had significantly longer stop-failure RTs than go RTs under both block types. In the group statistic for the “oculomotor only” condition, grand mean RTs of go trials (262.6 ± 10.5 ms) were shorter than grand mean RTs of stop failures (267.8 ± 9.4 ms), but this difference was not significant (*p* = 0.36). In the group statistic for the “manual only” condition, grand mean RTs of go trials for the hand responses (340.7 ± 9.3 ms) were longer than grand mean RTs of stop failures (324.9 ± 10.3 ms), and this difference was significant (*p* = 0.01). Also the biceps responses showed the same significant effect: Biceps reactions were significantly longer (285.9 ± 10.3 ms) than biceps responses of stop failures (271.8 ± 10.1 ms, *p* = 0.007). For the dual-response condition, grand mean eyes RTs in go trials (268 ± 8.5 ms) were shorter than stop failures (270.3 ± 7.1 ms), but this difference did not reach significance (*p* = 0.065). Both the mean response times of hands and muscles were significantly longer in the go than in the stop condition (hands go trials: 341.2 ± 8.7 ms vs. hands stop failures: 327.9 ± 11 ms, *p* = 0.016; muscles go trials: 286.6 ± 9.9 ms vs. muscles stop failures: 275 ± 18 ms, *p* = 0.012). No significant differences for saccadic RTs were found considering the saccadic responses of single and dual conditions together (go-trials RTs: 265.3 ± 6.5 ms vs. stop-failures RTs: 269.1 ± 5.6 ms, *p* = 0.293).

On a single subject basis, the analysis of eye RTs for stop trials and go trials showed a number of violations of the assumption of an independent race between go and inhibition processes. In particular, violations are more prevalent in case of fast reactions. In three participants, during the “oculomotor only” task, the differences between stop-failure RTs and go RTs reach significant values contrary to the first prediction, which assumes faster latencies for stop-failure RTs compared with go RTs. Importantly, the two participants with the slowest oculomotor go RTs, do not show violations. Apparently, only if the oculomotor RTs are fast enough, do mean go RTs and mean stop-failure RTs reveal a significant difference in direct opposition to the model's assumption. Such oculomotor violations are significant in the dual-response condition only for IM, who shows the fastest saccadic go RTs. On the other hand, mean saccadic go RTs for EH, and IW in the task “both” are longer than for the single-response task, whereas differences between go RTs and stop-failure RTs do not reach statistical significance. This reinforces the idea that interferences between go and inhibition processes do not occur in a longer time window.

***Preliminary conclusion*.** Biceps and hand RTs are consistent with the first model prediction. However, grand mean RTs for eye reactions failed to match this model prediction. For the saccadic reaction times, there are a number of violations of the first model prediction for some of the participants. Nevertheless, overall the violations did not provide strong evidence against this model prediction.

#### Testing the second prediction: stop-failure RTs increasing with SSD

**Figure 5:**
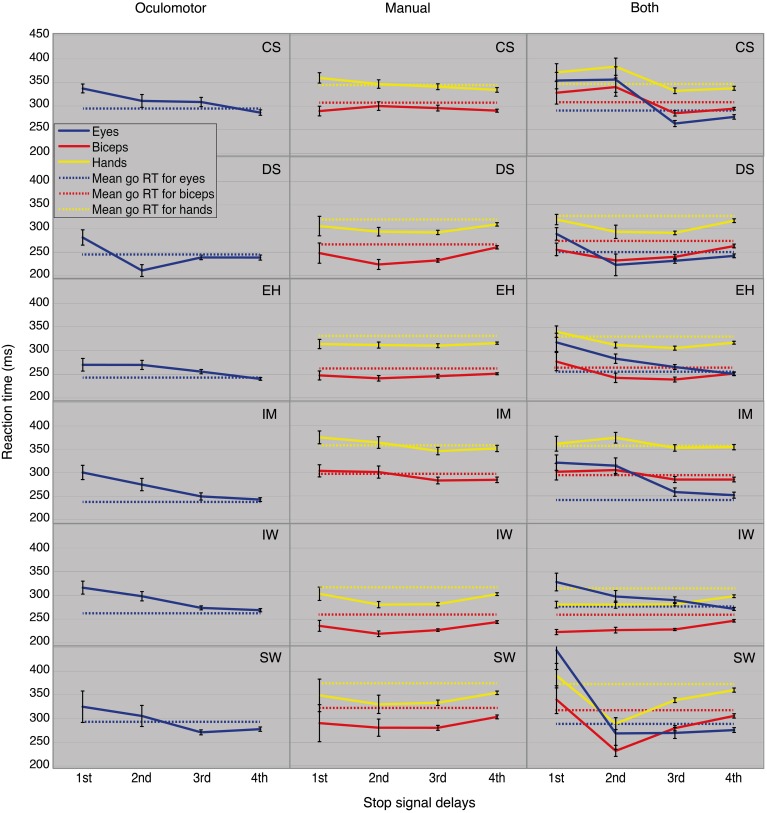
**Mean stop-failure RTs as a function of SSD for all conditions and participants**.

According to the model, the mean of the stop-failure RTs distribution should become longer for stop signals presented later (larger SSDs) and so approximating the mean of the go RTs distribution. Figure 5 depicts mean stop-failure RTs for all six participants (rows) and all block types (columns) and the corresponding go RTs. Stop-failure RTs for the saccadic eye movements systematically contradict this prediction. For participants CS, EH, IM, and IW, the mean stop-failure RTs for early SSDs are longer than for late ones. DS and SW show clearly longer stop-failure RTs only for the first SSDs, but a downward trend from the early to the late SSD still remains.

Stop-failure RTs for “manual only” are also not in accordance with the model predictions. No upward trend in the RTs is recognizable for successive SSDs. Instead, IM's and CS's hand and biceps RTs show a descending tendency through the SSDs. No violations of the second prediction were found for DS, IW, and SW from the second SSDs onward. The “both” condition shows these RT-trends contradict the model prediction more clearly. The oculomotor RTs at the first SSDs are always longer than at the successive SSDs. For the mean RTs of hands and biceps, the same effect can be observed for four of the six participants.

These trends were validated by *post-hoc* tests for go RTs and stop-failure RTs, grouped by SSDs (Table 5). For the first SSDs, five of the six participants show significantly longer oculomotor stop-failure RTs than go RTs. Only SW does not show a significant difference between first and mean go RT in the task “oculomotor only,” but there is still a clear downward trend in the SSDs (Figure 5, panel in the lower left corner). The right column in Table 5 summarizes the significant deviations from the model in detail.

**Table 5:** **Significance values of Bonferroni *post-hoc* tests**.

	**Dependent Variables**		**Independent variables**				**Multi-comparisons between SSD 1 2 3, and 4**
	**RTs of:**		**SSD 1**	**SSD 2**	**SSD 3**	**SSD 4**	***p* values against model-predictions**
CS	Ocular		**<0.001**	0.385	0.338	0.896	1 > 4 (<0.001)
	Manual	Hands	1.000	1.000	1.000	0.244	
		Biceps	1.000	1.000	1.000	0.087	
	Both	Eyes	**<0.001**	**0.030**	0.173	0.630	1 > 3 and 4(<0.001); 2 > 3 (0.001) and 4 (0.003)
		Hands	1.000	0.543	1.000	1.000	
		Biceps	1.000	**<0.001**	0.068	0.175	2 > 3 and 4 (<0.001)
DS	Ocular		**0.007**	0.004	1.000	1.000	1 > 2 (<0.001) and 3 (0.007) and 4 (0.004)
	Manual	Hands	1.000	0.733	<0.001	0.441	
		Biceps	0.003	0.161	0.002	1.000	
	Both	Eyes	**<0.001**	<0.001	0.325	1.000	1 > 2 and 3 and 4 (<0.001)
		Hands	0.003	0.312	<0.001	1.000	
		Biceps	0.002	0.196	0.007	1.000	
EH	Ocular		**0.007**	**<0.001**	0.051	1.000	1 > 4 (0.007); 2 > 4 (<0.001)
	Manual	Hands	0.611	0.047	<0.001	<0.001	
		Biceps	1.000	0.056	0.001	0.032	
	Both	Eyes	**<0.001**	**<0.001**	1.000	1.000	1 > 3 and 4 (<0.001)
		Hands	1.000	0.072	<0.001	0.005	
		Biceps	1.000	0.009	<0.001	0.036	
IM	Ocular		**<0.001**	**<0.001**	0.116	1.000	1 > 3 and 4 (<0.001); 2 > 4 (0.004)
	Manual	Hands	1.000	1.000	1.000	1.000	
		Biceps	1.000	1.000	0.458	1.000	
	Both	Eyes	**<0.001**	**<0.001**	0.938	1.000	1 > 3 and 4 (<0.001); 2 > 3 and 4 (<0.001)
		Hands	1.000	1.000	1.000	1.000	
		Biceps	1.000	1.000	1.000	1.000	
IW	Ocular		**<0.001**	**<0.001**	0.346	1.000	1 > 3 and 4 (<0.001); 2 > 4 (0.007)
	Manual	Hands	1.000	0.001	<0.001	0.127	
		Biceps	1.000	1.000	0.141	0.541	
	Both	Eyes	**0.005**	1.000	1.000	1.000	1 > 4 (0.001)
		Hands	0.154	0.066	<0.001	0.009	
		Biceps	1.000	1.000	<0.001	0.195	1 > 3 (0.040); 2 > 3 (0.050)
SW	Ocular		1.000	1.000	0.034	0.110	
	Manual	Hands	1.000	0.032	<0.001	0.004	
		Biceps	0.001	0.064	<0.001	0.014	
	Both	Eyes	**<0.001**	1.000	0.256	1.000	1 > 2 and 3 and 4 (<0.001)
		Hands	**0.015**	0.008	<0.001	0.794	1 > 2 and 3 (<0.001) and 4 (0.003)
		Biceps	0.426	<0.001	<0.001	1.000	1 > 2 (<0.001) and 3 (0.002)

On the left side, marked in bold, are the p values of the stop-failure RTs per SSD that were significantly longer than the go RTs. On the right side of the table, are the p values of the multiple comparisons between the stop-failure RTs per SSD. These are the cases violating the second prediction, that is, where the RTs for a preceding SSD were significantly longer than for a succeeding one.

A more fine-grained test of the effect of increasing SSD is given by a distribution inequality test implying a certain “fan pattern” among the stop-failure RT distributions and the go RT distribution (Osman et al., 1986; Colonius et al., 2001).

**Figure 6:**
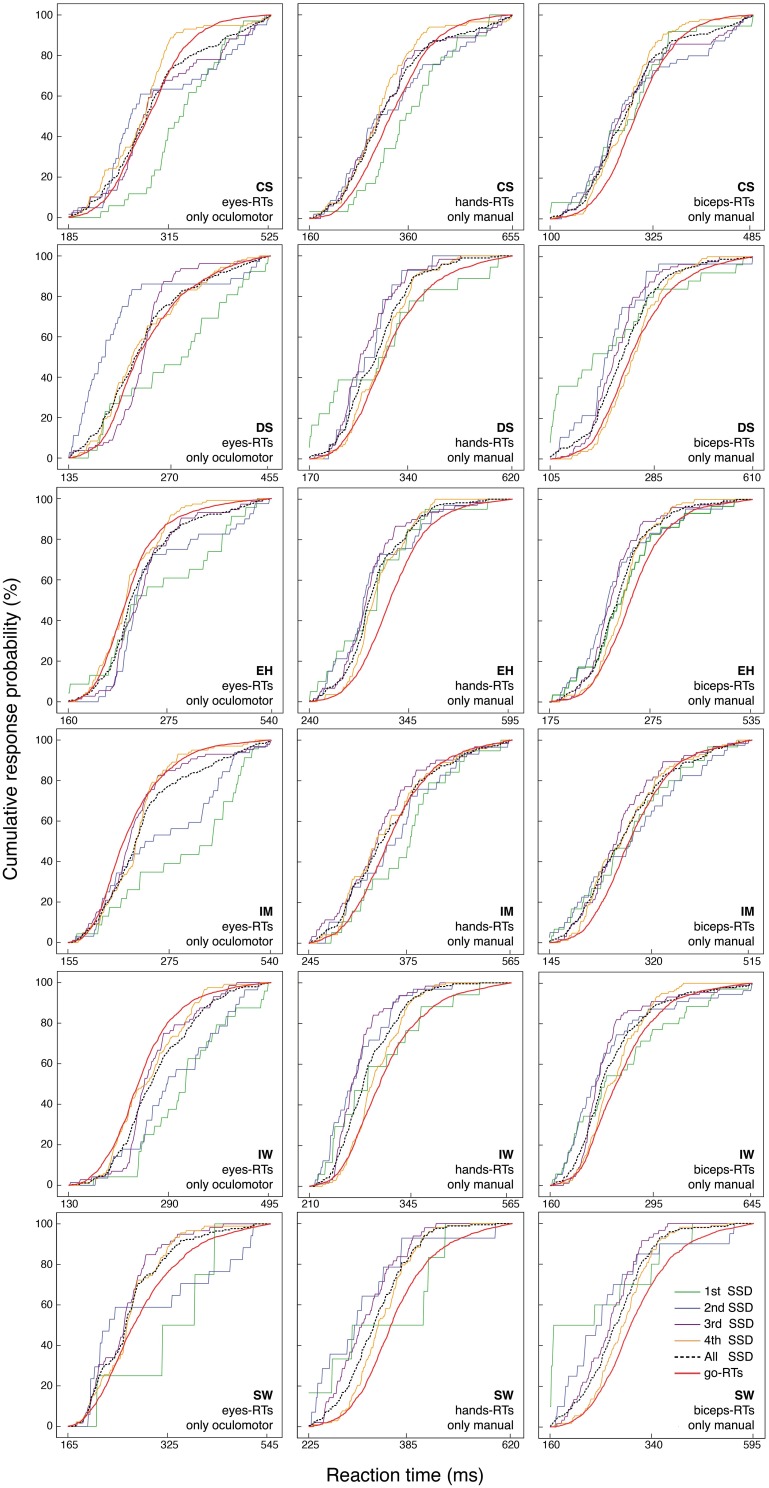
**Cumulative distribution functions of go RTs and stop-failure RTs for the four SSDs, separately for the eye, hand and biceps reactions**. The black dotted line represents the combination of all stop-failure RTs at the four SSDs.

As illustrated in Figure 6, the stop-failure RT distribution at the first SSD never matches the prediction for saccadic RTs for any participant, and there are many violations also for the second SSD. Some violations were also found for manual responses (for details, see the legend in Figure 6).

***Preliminary conclusion*.** Considering stop-failure RTs across SSDs reveals systematic violations of the second model prediction for all participants and in many of the conditions.

#### Testing the third prediction: predicting mean stop-failure RTs

For each SSD, mean stop-failure RT should be equal to the mean of those go RTs that are shorter than the estimated SSRT plus SSD. To test this prediction, we first need estimates of SSRT. Table 6 lists the SSRT estimates obtained using the integration method (see introduction). Over all 6 subjects, estimated SSRTs are in accordance with previous results (grand mean 153.5 9 ± 6.5 ms; Logan and Cowan, 1984; Hanes and Carpenter, 1999; Oezyurt et al., 2003; Akerfelt et al., 2006). SSRTs for eye movements are the shortest with a mean RT 119.9 ± 4.7 ms. The SSRTs of the hand are the longest (193.7 ± 7.9 ms), while biceps SSRTs have a mean value of 146.8 ± 8.1 ms. The SSRTs estimated for single-response and dual-response conditions are similar to each other (hand single-response: 193.1 ± 11.6 ms vs. dual response: 194.3 ± 11.9 ms, *p* = 0.728; biceps single-response: 148.2 ± 12.1 ms vs. dual response: 145.5 ± 11.9 ms, *p* = 0.156; eyes single-response: 117.8 ± 8.4 ms vs. dual response: 121.9 ± 4.9 ms, *p* = 0.420).

**Table 6:** **Estimated SSRT (ms) for inhibitory performances for eye, hand and biceps reactions**.

	**Task**		**SSD 1**	**SSD 2**	**SSD 3**	**SSD 4**	**Mean**
CS	Oculomotor		259	129	104	107	149.8
	Manual	Hands	307	182	165	154	202
		Biceps	280	152	138	127	174.3
	Both	Eyes	240	116	92	95	135.8
		Hands	303	180	160	163	201.5
		Biceps	277	151	126	130	171
DS	Oculomotor		206	130	96	96	132
	Manual	Hands	275	192	155	148	192.5
		Biceps	227	152	120	101	150
	Both	Eyes	202	134	99	87	130.5
		Hands	279	204	156	156	198.8
		Biceps	232	154	110	104	150
EH	Oculomotor		113	103	90	75	95.3
	Manual	Hands	200	190	176	171	184.3
		Biceps	140	127	114	114	123.8
	Both	Eyes	124	115	99	97	108.8
		Hands	202	190	175	171	184.5
		Biceps	139	127	110	103	119.8
IM	Oculomotor		176	132	97	95	117.5
	Manual	Hands	286	243	203	197	232.3
		Biceps	234	192	146	147	179.8
	Both	Eyes	181	137	102	106	131.5
		Hands	284	246	209	211	237.5
		Biceps	230	192	148	149	179.8
IW	Oculomotor		143	103	79	67	98
	Manual	Hands	179	151	128	123	145.3
		Biceps	134	109	89	80	103
	Both	Eyes	144	119	98	85	111.5
		Hands	174	154	144	118	147.5
		Biceps	132	109	102	72	103.8
SW	Oculomotor		176	110	87	84	114.3
	Manual	Hands	270	188	171	179	202
		Biceps	223	141	126	143	158.3
	Both	Eyes	180	103	85	86	113.5
		Hands	263	192	169	159	195.8
		Biceps	214	148	119	113	148.5

Table 7 presents the predicted vs. observed mean stop-failure RTs for saccadic, manual and biceps reactions separately for each SSD as well as averaged across SSDs for all participants. In 94% of all cases, predicted means are shorter than the recorded ones. The largest discrepancies occur for the values of the first SSD of each reaction type. Moreover, for the oculomotor task, observed mean stop-failure RTs decrease with SSD, whereas the prediction is in the opposite direction.

**Table 7:** **Mean stop-failure RTs for each SSD and weighted-average across all SSDs**.

	**Stop RTs (ms) of task:**		**SSD 1**	**SSD 2**	**SSD 3**	**SSD 4**	**Average**
		**Emp./Est**.	**Emp./Est**.	**Emp./Est**.	**Emp./Est**.	**Emp./Est**.
CS	Ocular		**337.2**/238.4	**310.8**/244.8	**308.5**/253.6	**286.1**/276.3	**302.5**/260.0
	Manual	Hands	**359.6**/291.0	**346.6**/301.1	**340.9**/314.3	**334.1**/328.7	**340.9**/315.8
		Biceps	**300.6**/258.6	**305.6**/268.0	**303.1**/282.9	**294.3**/294.1	**299.6**/281.6
	Both	Eyes	**349.1**/221.5	**317.0**/233.5	**271.7**/245.4	**279.3**/269.8	**289.2**/253.9
		Hands	**362.8**/287.8	**360.4**/301.3	**336.2**/313.4	**338.1**/332.7	**343.1**/318.7
		Biceps	**313.3**/258.7	**337.1**/268.3	**293.4**/277.3	**297.3**/296.4	**305.4**/281.8
DS	Ocular		**280.9**/188.6	**210.9**/191.9	**238.7**/213.0	**238.4**/234.2	**238.9**/216.4
	Manual	Hands	**305.1**/258.7	**293.2**/256.5	**291.6**/284.1	**308.7**/308.3	**301.9**/292.4
		Biceps	**225.0**/207.4	**239.5**/211.1	**242.0**/237.7	**261.7**/255.2	**248.8**/238.8
	Both	Eyes	**306.5**/188.1	**206.8**/197.5	**234.9**/217.2	**242.0**/235.7	**240.9**/220.0
		Hands	**281.7**/264.2	**293.6**/267.8	**292.4**/287.3	**317.6**/315.0	**305.3**/297.5
		Biceps	**231.4**/212.8	**245.8**/214.4	**253.1**/239.1	**265.1**/263.2	**256.3**/245.9
EH	Ocular		**270.0**/203.2	**269.8**/210.1	**255.5**/220.2	**240.2**/232	**252.3**/222.4
	Manual	Hands	**313.9**/286.5	**311.7**/294.5	**310.3**/304.9	**315.7**/322.5	**313.5**/310.1
		Biceps	**254.8**/225.6	**246.9**/230.5	**246.5**/241.5	**252.7**/257.2	**250.3**/244.9
	Both	Eyes	**318.4**/213.1	**289.9**/221.0	**266.1**/229.4	**252.3**/246.6	**266.9**/234.9
		Hands	**332.2**/289.4	**312.9**/294.8	**305.4**/304.9	**318.3**/322.5	**315.5**/310.2
		Biceps	**266.7**/226.4	**245.0/**231.9	**243.8**/240.7	**253.8**/256.2	**250.9**/244.8
IM	Ocular		**301.7**/195.8	**275.8**/200.5	**250.5**/217.6	**243.7**/223.2	**255.9**/215.7
	Manual	Hands	**358.9**/296.2	**364.0**/300.8	**345.8**/320.9	**351.4**/328.5	**354.0**/317.9
		Biceps	**298.1**/243.0	**295.5**/248.6	**283.5**/263.2	**287.9**/272.8	**288.3**/261.5
	Both	Eyes	**319.1**/201.7	**294.9**/206.2	**253.0**/222.3	**251.3**/229.2	**263.8**/221.0
		Hands	**360.8**/296.8	**368.4**/305.4	**353.2**/325.3	**355.4**/336.0	**357.1**/324.0
		Biceps	**308.3**/239.2	**288.5**/249.3	**285.6**/264.4	**291.1**/274.8	**290.6**/263.1
IW	Ocular		**316.4**/217.2	**298.2**/218.1	**273.7**/235.7	**268.8**/253.1	**278.3**/240.3
	Manual	Hands	**303.5**/255.3	**280.6**/264.8	**281.6** /280.2	**302.6**/299.9	**293.3**/258.5
		Biceps	**262.6**/209.1	**254.1**/217.6	**243.2**/232.0	**248.0**/247.8	**249.4**/233.2
	Both	Eyes	**326.1**/220.4	**294.9**/232.7	**289.4**/251.7	**272.5**/268.9	**283.4**/256.2
		Hands	**281.0**/253.5	**291.1**/268.2	**285.6**/289.9	**299.8**/302.4	**292.9**/291.1
		Biceps	**271.0**/209.2	**263.0**/221.7	**238.1**/241.7	**250.1**/250.9	**249.4**/240.1
SW	Ocular		**324.8**/207.6	**305.4**/224.9	**270.9**/247.9	**277.5**/267.4	**279.1**/255.0
	Manual	Hands	**348.7**/291.8	**329.9**/299.2	**333.1**/330.1	**354.2**/355.8	**346.0**/339.7
		Biceps	**253.3**/244.5	**287.2**/251.2	**285.3**/281.7	**304.7**/307.1	**294.6**/289.4
	Both	Eyes	**443.7**/208.7	**263.2**/220.2	**271.1**/244.8	**278.6**/266.4	**277.9**/253.2
		Hands	**444.5**/285.3	**324.7**/301.2	**339.9**/327.1	**360.3**/349.1	**353.3**/333.9
		Biceps	**361.3**/236.8	**265.7**/253.4	**284.1**/275.7	**310.1**/297.1	**298.5**/280.9

On the left in bold are the empirically observed (Emp.) stop-failure RTs, on the right the estimated (Est.) stop-failure RTs according to the third prediction. Stop-failure RTs were calculated on the basis of the go RTs distribution and by using the inhibition probability of the single SSDs. Underlined are the few cases for which the estimated stop-failure RT was longer than the observed one.

***Preliminary conclusion*.** In general, predicted mean stop-failure RTs are too small. While this may be interpreted as evidence against the model assumption, it may at least in part be a due to our using the integration method for estimating SSRT (see discussion below).

### Interaction of concomitant eye and hand movements

Mean saccadic latencies in go trials range between 240 and 300 ms (grand mean 265.3 ± 6.5 ms). Pointing latencies are on average 75.7 ms longer than saccadic latencies. The biceps muscle activation onset precedes the onset of the pointing movements on average by 54.7 ms. The latencies under single-response and dual-response conditions are listed in Table 8 for each participant individually and as grand means.

**Table 8:** **Comparison of mean latencies in single-task vs. dual-task conditions for eye and pointing movements and for biceps activity in trials where no stop signal was presented**.

	**Eyes**			**Hands**			**Biceps**			
	**Mean RT (ms):**		***U*-test**	**Mean RT (ms):**		***U*-test**	**Mean RT (ms):**			***U*-test**
	**Single**	**Dual**	***p*(2-tailed)**	**Single**	**Dual**	***p*(2-tailed)**	**Single**	**Dual**		***p*(2-tailed)**
CS	294.4 > 289.6		0.001	344.5 < 345.9		n.s.	306.3 < 308.1			n.s.
DS	243.9 < 249.1		n.s.	**320.3 < 326.0**		**<0.001**	**265.7 < 272.8**			**<0.001**
EH	**242.6 < 257.2**		**<0.001**	331.0 < 331.4		n.s.	263.2 < 264.2			n.s.
IM	**238.1 < 242.7**		**<0.001**	358.9 > 357.8		n.s.	298.1 > 293.8			n.s.
IW	**263.2 < 280.0**		**<0.001**	315.5 > 314.6		n.s.	259.3 < 261.0			0.004
SW	293.7 > 289.5		n.s.	374.3 > 371.7		n.s.	323.1 > 319.7			n.s.
	**Mean RT (ms):**		***T*-test**	**Mean RT (ms):**		***T*-test**	**Mean RT (ms):**			***T*-test**
	**Single**	**Dual**	***p*(2-tailed)**	**Single**	**Dual**	***p*(2-tailed)**	**Single**	**Dual**		***p*(2-tailed)**
GM	262.7 < 268.0		n.s.	340.8 < 341.2		n.s.	286.0 < 286.6			n.s.

Marked in bold are the values, where participants responded significantly faster in the single-task than in the dual-task condition.

Mean saccadic latencies in go trials differ between single-response and dual-response conditions for 4 out of 6 participants. Four participants responded significantly faster in the single-response condition, one participant (CS) significantly faster in the dual-response condition, and for one participant (SW) no significant differences were found (2-tailed Mann-Whitney *U* test in Table 8). In contrast to the saccadic latencies, the response times of pointing movements and related biceps activities mostly did not differ significantly between single-response and dual-response tasks. Only three of the twelve data sets, namely hand and biceps of participant DS and biceps of participant IW, show significant differences between single-response and dual-response conditions (see Table 8). In all significant cases latencies are smaller in the single-response condition (2-tailed Mann-Whitney *U* test in Table 8). Grand mean latencies of dual-response conditions are always longer than grand mean latencies of single-response conditions, even if this difference is not significant.

In the dual-response condition, go-trial latencies of concomitant saccades and pointing movements show significant, low to moderate correlations (Table 9). In contrast, the correlation between biceps activity onset and pointing onset is high. Correlations values between latencies of erroneous eye and hand responses in stop failures are lower than the corresponding values in go trials. Nevertheless the comparison between these two groups narrowly missed significance (*p* = 0.052). For two participants these correlations were not significant. Also correlations values between erroneous hand and biceps onsets are lower than in go trials, however a comparison between these two groups missed significance (*p* = 0.096).

**Table 9:** **Pearsons's correlations between pointing and saccadic latencies and between pointing and biceps latencies for go trials and stop trials, individually for each participant**.

	**Go trials**				**Stop failures**			
	**Eye-hand**		**Hand-biceps**		**Eye-hand**		**Hand-biceps**	
CS	0.428	*p* < 0.000	0.855	*p* < 0.000	0.307	*p* < 0.000	0.828	*p* < 0.000
DS	0.456	*p* < 0.000	0.895	*p* < 0.000	0.290	*p* < 0.000	0.817	*p* < 0.000
EH	0.622	*p* < 0.000	0.953	*p* < 0.000	0.552	*p* < 0.000	0.948	*p* < 0.000
IM	0.452	*p* < 0.000	0.905	*p* < 0.000	0.420	*p* < 0.000	0.755	*p* < 0.000
IW	0.695	*p* < 0.000	0.962	*p* < 0.000	0.087	n.s.	0.271	*p* < 0.000
SW	0.477	*p* < 0.000	0.935	*p* < 0.000	0.080	n.s.	0.242	*p* < 0.000

## Discussion

This study presents stopping behavior of six participants measuring saccadic eye movements, finger pointing movements, and muscle (biceps) activation. Each participant underwent extensive training (10–12 sessions) and was subsequently tested in three output conditions, “oculomotor only,” “manual only,” or “both,” totaling in more than 6000 measurements per person. This setup provided various opportunities to study mechanisms of inhibition. First, potential violations of the independence assumptions of the horse race model could be tested more thoroughly than in previous studies since more observations of stop-failure RTs were available. Second, measuring concomitant saccadic and hand movements in both dual and single-task conditions allowed to probe possible interactions between these differing modes of inhibitory behavior. Third, registering arm muscle activity delivered information about the cancellation of motor commands even when no open movement could be observed.

### Consequences from the horse race model tests

Concerning the independence of stop and go-signal processing, the test of the second model prediction revealed that only the fastest, unsuccessfully inhibited saccadic reactions, escaping the first SSD, showed effects of this interaction, whereas the slower hand and biceps stop-failure RTs generally matched the model predictions. Thus, our data supports the suggestion that the unsuccessfully inhibited saccadic reactions compete with the stop-signal processing for common resources (Oezyurt et al., 2003; Akerfelt et al., 2006). Whether this competitive interaction occurs from the very beginning of the “race” or only later, as proposed by the interactive race model of Boucher et al. (2007a), remains open at this point. Moreover, in Boucher et al. (2007b) the existence of longer than expected stop failures at short SSDs is attributed to subjects not having failed to inhibit movement but subsequently making the movement, possibly as a result of subjects' impatience in maintaining fixation for the duration of the trial (ibid, p. 800). We cannot rule out this potential explanation for our data altogether, although there are several subject/SSD conditions where the length of SSRT+SSD compared to mean stop-failure RT (233 ms vs. 278 ms for Subject IW, e.g.) makes this explanation unlikely.

RTs ascending with SSD, as predicted by the model, were not observed for hand and biceps reactions, either. We would expect that the fastest hand reactions also compete with the stop-signal processing for resources. Consequently, more violations would also be expected for quick biceps responses. Longer latencies at the first SSD, compared to successive SSDs, for the biceps stop-failure RTs would fit the previous results. Indeed, there are general violations of the second prediction by most of the participants (see Figure 5). Nevertheless, relevant deviations from the values predicted by the model regarding biceps stop-failure RTs in comparison with slower hand stop-failure RTs, have not been found. Thus, it seems that the fastest biceps reactions, although are shorter than the hand RTs, do not compete with the hand reactions for limited common resources.

Using the integration method to estimate SSRT, predicted mean stop-failure RTs were found to be too small under nearly all conditions, in contrast to the third model prediction. While this may be interpreted as additional evidence against the independence assumption, it has been argued, based on simulations performed in Band et al. (2003), that this may be due to the assumption of constant SSRT on which the integration method is founded (cf. Verbruggen and Logan, 2009; p. 654; Verbruggen et al., 2013).

For future studies, one should note that up until recently, the only existing method for estimating the entire distribution of SSRT was proposed by Colonius (1990; see also De Jong et al., 1990; p. 181). However, as shown by simulations (Band et al., 2003) the resulting estimates are not precise enough under the limited number of observations that are typically available. Matzke et al. (2013) developed an alternative yielding an estimate of the entire SSRT distribution based on Bayesian parametric estimation and relying on Markov chain Monte Carlo sampling to obtain posterior distributions for the model parameters. In its current form, this Bayesian parametric approach assumes go RTs and SSRTs are ex-Gaussian distributed. Other recent alternatives for estimating SSRT rely on other specific parametric models (Kornylo et al., 2003; Boucher et al., 2007a; Salinas and Stanford, 2013; Logan et al., 2014).

### Longer latencies in stop-signal experiments

The difference between manual and oculomotor latencies corresponds to findings reported in other studies (Fischer and Rogal, 1986; Bekkering et al., 1994). In general, latencies in stop-signal experiments are slightly longer than in mere goal-directed time tasks under comparable stimulus conditions (Oezyurt et al., 2003). One possible explanation is the participants' strategy to delay the response in order to reduce the probability of erroneous responses in case of stop trials (cf. Bissett and Logan, 2012a,b). Although our participants were extensively trained to respond as fast as possible, this effect may not have been completely suppressed. The rates of correctly withheld responses in stop trials corroborate this interpretation^4^. These data suggest a trade-off between inhibition probability and RT. On the other hand, saccadic response latencies increase after successfully canceled stop trials (Emeric et al., 2007). These results offer an alternative explanation for longer latencies in stop-signal experiments.

### Inhibition probabilities for manual and ocular reactions

Given the large difference between oculomotor and hand movement latencies, one could have expected inhibition probabilities to differ markedly for a given SSD: The longer latencies of arm movements may give more leeway for stopping processes. However, the finding that inhibition probabilities are in the same range and not systematically higher for arm movements suggests that detection and processing time of the stop signal may be identical for both movements. Note that estimated SSRTs with a mean value of 150 ms are clearly shorter than any mean RT regardless of effector type and condition. This indicates a common input and processing pathway for stop-signal detection but two different outcomes for eye and hand RTs. They show the same inhibition probability due to the short SSRTs of the central selective inhibition mechanism. At the same time, eye reactions also show evidence of stop-signal interferences at the shortest SSDs, probably due to the absence of an effective peripheral stopping mechanism. There is also a possible conflict for common resources, because the saccadic RTs are closer to the SSRTs.

The individual stop probability for each effector does not change in the dual-task in comparison to the single-task condition. This reinforces the hypothesis that participants adapt their latencies to the task affordances so that their individual probability to withhold the response remains stable under different conditions. Inhibition probability for biceps activation is always smaller than for pointing movements. This indicates a peripheral inhibition of the pointing movement, probably by activation of muscle antagonists (De Jong et al., 1990).

### Saccades in stop trials are hypometric

The tendency toward reduced correlation between pointing and biceps activity in stop trials compared with go trials possibly indicates that inhibitory processes may affect pointing and biceps activity differently. We assume that several muscles are involved in peripheral response suppression. These muscles affect the hand movement but not the activation of the biceps. While peripheral inhibition is conceivable for limb movements, equivalent processes are less obvious for saccadic eye movements, which are typically considered to be ballistic. This would make a peripheral inhibition of saccades impossible. However, we find that saccades in signal response trials are hypometric. Saccadic amplitudes are smaller in stop trials than in go trials. This finding is in line with the results of other studies (Oezyurt et al., 2003; Akerfelt et al., 2006; Walton and Gandhi, 2006). The execution of saccadic eye movements is altered by the stop signal. We suggest that the stop signal induces inhibitory processes in the saccadic system, but they are not strong enough to suppress the response completely. Conflicts between go and stop signals for limited common resources take place in an early (central) processing stage before an output is reached, resulting in a delayed and hypometric oculomotor response.

### Independence of the effectors

Eye and hand movement errors in go trials under dual-response conditions suggest that the movements are performed independently from each other. Direction errors, as well as erroneous stopping of the movements, do not depend on the responses given with the other effector. This hints at an independent preparation of spatial movement parameters (see also Mirabella et al., 2006). The correlation between eye and hand movement latencies in go trials is in the range reported in other studies (Logan and Irwin, 2000; Boucher et al., 2007b). These low to medium correlations support the notion of independent movement preparation by common sensory or attentional processes (Bekkering et al., 1995; Hodgson et al., 1999; Yamaguchi et al., 2012). The high correlation between onsets of pointing and biceps muscle activity is expected given the biceps is involved in lifting the hand and forearm during the pointing movement.

The processes of countermanding concomitant saccades and pointing movements seem also to be independent. The occurrence of stop trials, in which only one movement is inhibited whereas the other is executed, provides additional evidence for this assumption. This is corroborated by the correlation between eye and hand movements in those trials in which both movements are executed. The correlations show a tendency to be smaller in stop trials than in go trials. This suggests that the process of inhibiting the response may add further variability due to possible additional processing steps that differ for oculomotor and arm movement control. Nevertheless, these hints should be considered carefully, since many subjects still show moderate eye-hand correlations in both go trials and stop failures in the dual condition. The observed variability in the correlation analysis may have been introduced by the different inhibition functions for eyes and hands: at the same SSD eyes and hands may have been stopped more or less efficiently, thus adding variability in these correlations.

### Evidence of a double inhibition system

In their model for the control of limb movements, Bullock and Grossberg (1988, 1991) suggested that central processes are involved in the programming of movement direction and amplitude, whereas peripheral processes control movement onset and speed. Accordingly, De Jong et al. (1995) differentiate two inhibitory mechanisms: The first is described as central and operates by inhibiting response activation processes in cortical motor structures, therewith preventing the central outflow of motor commands. The other mechanism is described as peripheral and prevents the actual execution of central motor commands by peripheral motor structures, possibly by blocking the transmission of such commands. Consequently, the peripheral mechanism can achieve fast motor inhibition by stopping the central outflow “downstream.”

Specifically, in the single-response as well as in the dual-response task, participant IW always showed quicker biceps RTs than the corresponding oculomotor RTs (Figure 5). This corroborates the two-mechanism model: When significant interferences between the oculomotor reactions and inhibition processes at the first SSDs were found, similar interferences for the even faster biceps reactions could not be observed or only weakly in the task “manual only”. This could be due to the quick activation of a highly efficient peripheral inhibitory mechanism (De Jong et al., 1990). This peripheral inhibitory mechanism is, however, probably ineffective for saccades (Boucher et al., 2007b).

On the other hand, a recent transcranial magnetic stimulation (TMS) study by Wessel et al. (2013) found that saccade suppression may have global motor effects: 50 ms before the estimated time at which a saccade is successfully stopped there was a reduced corticospinal excitability for the hand, as measured by TMS of the motor cortex (M1) (for a similar result, see also Badry et al., 2009). Whether a similar effect occurs in the dual-task condition (stopping eye and hand) seems not clear yet.

## Concluding remarks

The current study corroborates previous evidence pointing to an interaction occurring between the processing of the go and the stop signal (Oezyurt et al., 2003). Due to the fact that the number of observations for short SSDs is larger in this study than in previous studies, this conclusion could be supported by statistical analysis for the saccadic responses. The interactive race model by Boucher et al. (2007a) takes this dependency into account by assuming a brief but potent interaction effect between the stop-signal and the go-signal processes after a period of independent processing. According to the authors, SSRT then primarily reflects the period before the stop unit is activated, during which stop and go processing are independent. Recent results by Nelson et al. (2010) demonstrate the robustness of the race model regarding this non-independence and, moreover, regarding the non-stationarity of reactions across trials which has also been observed in other studies (e.g., Emeric et al., 2007; Corneil et al., 2013). Nevertheless, it has recently been shown that SSRT may also be subject to strategic and motivational influences (e.g., speed-accuracy trade-off) and that additional measures may be called for (Leotti and Wager, 2010). Furthermore, fatigue and attention may also play an important role for the stop-failures distribution: Due to fatigue or reduced attention, subjects could be less efficient in detecting stop signals in coming trials. If some stop failures are caused by inattention or drowsiness, these trials may represent a relevant proportion of stop failures in particular at shorter SSDs, thus resulting in an apparent violation of the race model for responses escaping early stop signals.

Violations of the race model predictions, as evidenced for saccadic responses, were not found for hand and biceps reactions, which in contrast satisfy the model predictions. An explanation for this discrepancy between effectors could be that longer RTs (as typical for hand and biceps reactions in comparison to saccadic responses) did not conflict with the much faster inhibition process. An alternative explanation draws upon the hypothesis of an efficient peripheral inhibitory mechanism. Through EMG-recording of the *biceps brachii*, we could observe muscle activity of the successfully inhibited hand movements. By activating antagonist muscles according to De Jong et al. (1990), hand reactions could still be stopped in the very last moment, giving a one/zero response just as expected by the race model. On the other hand, fast eye movements like saccades can be slowed down only through the viscosity of the bulb. Our data support this second explanation since, whenever the biceps and hand RTs were faster than the concomitant oculomotor reactions, no significant interference for hand and biceps RTs was found, just as for oculomotor RTs. Our measurements of biceps activity complements work by Goonetilleke et al. (2010, 2012) recording antagonist neck muscle activity during a head-unrestricted oculomotor countermanding task. The timing of the burst relative to the stop signal (the antagonist muscle latency) correlated positively with estimates of the SSRT, even though antagonist muscle latencies were about 50 ms longer. These authors interpret these observations as being consistent with the hypothesis that antagonist muscle recruitment arises as a peripheral manifestation of oculomotor cancellation, with longer antagonist muscle latencies arising from the efferent delay from cancellation of the oculomotor program to the onset of antagonist muscle recruitment.

Our findings for the dual-response task indicate that concomitant eye and hand movements are not coupled with respect to the preparation or the inhibition process. This provides strong evidence for largely independent control processes for the two effectors, in spite of common sensory processing of go and stop signals, similar to findings of a recent study with combined eye and head movements (Corneil and Elsley, 2005). For future studies, it would be interesting to extend our dual-task in such a way that participants' foreknowledge about which response (hand or eye) to inhibit is varied. Recent results revealed that in such a case selective and global mechanisms of stopping could be dissociated (Aron and Verbruggen, 2008; see also Ko and Miller, 2013; Bissett and Logan, 2014).

## Author contributions

The work presented here was carried out in collaboration between all authors. Alessandro Gulberti, Petra A. Arndt and Hans Colonius defined the research theme. Petra A. Arndt and Hans Colonius designed methods and experiment. Alessandro Gulberti carried out the laboratory experiments, analyzed the data, interpreted the results and wrote the paper. Petra A. Arndt and Hans Colonius discussed analyses, interpretation, and presentation. All authors have contributed to, seen and approved the manuscript.

### Conflict of interest statement

The authors declare that the research was conducted in the absence of any commercial or financial relationships that could be construed as a potential conflict of interest.

## Footnotes

^1^Because there is no observable reaction involved, a more fitting name would be stop-signal processing time (SSPT) but this term has not found wide-spread usage in the literature.

^2^Response rate: the proportion of stop trials in which responses are given (i.e., failed inhibitions).

^3^The one-tailed *t*-test is performed since we specifically intended to test if the stop signal had an inhibiting effect on the saccadic amplitudes of stop failures, as clearly demonstrated in the ANOVA on a single participants basis.

^4^In the condition block “oculomotor only,” participants CS and SW show the longest saccadic RTs in go trials (45 ms longer than the average), and they have the best inhibition probabilities for saccades (CS = 57.5%; DS = 49.1%; EH = 51.8%; IM = 52.0%; IW = 57.5%; SW = 66.1%; see also Figure 3). In case of pointing latencies for the condition block “manual only,” IM and SW react the slowest in trials where no stop signals were presented (25 ms longer than the average, see Table 4), and these two participants have the best inhibition probabilities for pointing movements (CS = 54.6%; DS = 54.6%; EH = 54.0%; IM = 62.0%; IW = 56.0%; SW = 64.8%; see also Figure 3).
